# Incidence and risk factors of immune‐related adverse events induced by immune checkpoint inhibitors among older adults with non‐small cell lung cancer

**DOI:** 10.1002/cam4.6879

**Published:** 2024-01-02

**Authors:** Yiran Rong, John P. Bentley, Kaustuv Bhattacharya, Yi Yang, Yunhee Chang, Sally Earl, Sujith Ramachandran

**Affiliations:** ^1^ Department of Pharmacy Administration University of Mississippi University Mississippi USA; ^2^ Center for Pharmaceutical Marketing and Management Center for Pharmaceutical Marketing and Management University of Mississippi University Mississippi USA; ^3^ Department of Nutrition and Hospitality Management University of Mississippi University Mississippi USA; ^4^ Department of Pharmacy Practice University of Mississippi University Mississippi USA

**Keywords:** immune checkpoint inhibitors, immune‐related adverse events, non‐small‐cell lung cancer, older adults, SEER‐Medicare

## Abstract

**Background:**

Immune checkpoint inhibitor (ICI) treatment has been linked to a variety of immune‐related adverse events (irAEs), which can affect any organ system. The incidence and risk factors of irAEs have not been adequately evaluated among older adults with NSCLC.

**Methods:**

A cohort study was conducted using 1999–2019 SEER‐Medicare data among beneficiaries aged ≥65 years with a diagnosis of NSCLC who received nivolumab, pembrolizumab, or atezolizumab. Incident irAEs were identified post‐ICI initiation. Demographic, cancer‐related characteristics, and clinical history risk factors of irAEs were evaluated with competing events considered.

**Results:**

A total of 8175 older NSCLC patients were included (with 46.8% experiencing irAEs). Pneumonitis (16.5%), hypothyroidism (10.5%), arrhythmia (11.18%), and acute kidney injury (AKI) (5.8%) were the most common irAEs. The median time to first irAE was 82 days (IQR: 29–182 days). The earliest onset of irAE occurrence was for hematologic irAEs, while the latest were gastrointestinal, dermatologic, and musculoskeletal irAEs. Fine–Gray regression modeling revealed significantly greater hazards of irAE occurrence in patients who received pembrolizumab at index, did not have CNS metastases, had a history of autoimmune disorder, and had chemotherapy in combination with ICI. Race, socioeconomic status, previous radiation therapy, and comorbidity burden were found to be associated with the occurrence of certain type of irAEs.

**Conclusion:**

A significant proportion of older patients with NSCLC develop an irAE after receiving ICI treatment. Factors related to cancer and treatment as well as demographics contribute to the increased risk of irAEs. Close monitoring and prediction of irAE among older patients receiving ICI is warranted.

## 1 INTRODUCTION

Immune checkpoint inhibitors (ICIs) have rapidly become the new standard of care for both first‐ and second‐line treatment of advanced non‐small‐cell lung cancer (NSCLC).^1^, ^2^ The efficacy and effectiveness of ICIs has been equally demonstrated among older and younger adults with NSCLC.^3^, ^4^, ^5^, ^6^, ^7^, ^8^ However, ICIs can induce autoimmune‐like adverse events that affect almost any organ or organ system, which are termed immune‐related adverse events (irAEs).^9^, ^10^, ^11^, ^12^ The general incidence of irAEs was reported to be 20%–45% in randomized controlled trials (RCTs)^12^, ^13^ and 33%–50% in observational studies for any grade.^7^, ^14^, ^15^, ^16^, ^17^ Most irAEs are mild to moderate in severity^18^; however, fatal irAEs are reported in 0.3%–1.3% of treated patients, with a majority being myocarditis, followed by pneumonitis, hepatitis, myositis, nephritis, neurologic, and hematologic adverse effects.^19^

Despite a growing body of research focused on ICI‐induced irAEs, the toxicity profile in older patients with NSCLC remains unclear. Older adults are usually underrepresented in RCTs due to age restriction, the presence of comorbidities, poor performance status, or lack of sufficient social support.^20^ Only 31.7%–41.0% of RCT participants are older than 65 years, while more than 60% of patients with NSCLC who received ICIs in real‐world practice are older than 65.^21^ Moreover, existing observational studies targeting older adults with NSCLC are limited by small sample sizes,^6^, ^7^, ^15^ short follow‐up time,^6^, ^7^ or single ICI regimens.^15^, ^22^ The occurrence of irAEs may result in treatment discontinuation, as well as morbidity and mortality.^23^, ^24^, ^25^ The management of irAEs for older patients is further challenged by potential comorbidity, frailty, dysfunction of organs, and polypharmacy.^20^, ^25^ It is necessary to understand the nature, characteristics, and impact of irAEs for older NSCLC patients undergoing ICI therapy in routine practice at a population level. This population‐based study aimed to characterize the incidence and clinical features of ICI‐induced irAEs in a large cohort of older adults with NSCLC. Demographic, cancer‐related characteristics, and clinical history risk factors of irAE incidence are also evaluated.

## 2 METHODS

### 2.1 Study design and data source

Using the Surveillance, Epidemiology, and End Results (SEER)‐Medicare linked database, a cohort study was conducted to characterize features of irAEs as well as assess their risk factors among Medicare beneficiaries with NSCLC treated with ICIs. The SEER database collects newly diagnosed cancer patients' data from 21 cancer registries across various geographical areas of the United States covering approximately 34.6% of the US population. The SEER‐Medicare linked database comprises population‐based data of cancer patients in the SEER program and their matched Medicare administrative claims data, which allows a longitudinal assessment of diagnoses, treatments, and service use before and after the cancer diagnosis. This study used the 2020 linkage dataset, which includes 1999–2017 SEER data linked with 1999–2019 Medicare claims data. This study was approved by the Institutional Review Board at the University of Mississippi (protocol #21–036).

### 2.2 Study population

This study included Medicare beneficiaries who entered the SEER registry from 1999 through 2017, were diagnosed with NSCLC as primary site of cancer, received ICI treatment, and were at least 65 years of age at their first diagnosis with NSCLC based on International Classification of Diseases for Oncology, third edition (ICD‐O‐3) codes. Patients were excluded if the diagnosis of NSCLC was made by death certificate or autopsy. Patients were identified as having received ICI therapy if they received at least one cycle of either pembrolizumab, nivolumab, or atezolizumab from March, 2015 (the time that the first ICI agent was approved by the FDA for treating NSCLC) to December, 2018, either as monotherapy or combined with chemotherapy or other anticancer agents. Healthcare Common Procedure Coding System (HCPCS) codes (C9453 and J9299 for nivolumab, C9027 and J9271 for pembrolizumab, and C9384 and J9022 for atezolizumab) were used to identify the administration of ICIs. The date of medical claim indicating the first dose of ICI administration defined the index date for each patient. To ensure that patients were incident users of ICIs for treating NSCLC, we excluded those who had any use of ICIs prior to March, 2015. Continuous enrollment in Medicare Part A, Part B, and Part D was required from the month of diagnosis or for at least 12 months before the first dose of ICI, whichever was longer, through the index date. Patients were excluded if they were found to be enrolled in a health maintenance organization (HMO) in the pre‐ICI initiation period. The model treated disenrollment from Medicare Part A, B, and D coverage, enrollment in HMO, switching to other ICI agents, or reaching the end of the study period without an event (December, 2019) as censored.

### 2.3 Outcome of interest

The outcome variable for this study was the incident occurrence of irAEs post the initiation of ICI treatment. Immune‐related adverse events are defined as unique adverse events that have an autoimmune basis requiring more frequent monitoring and potential intervention with immune suppression therapy.^26^ In this study, an irAE was defined as present if a patient had a new medical claim diagnosis (no same event occurred within 12 months before the onset of irAE) with a corresponding irAE diagnosis code^16^ between the index date and the 90th day post the last dose of ICI. The list of irAEs for this study was compiled based on clinical guidelines, clinical opinion, previous studies, and clinical trials. International Classification of Diseases, Ninth Revision, Clinical Modification (ICD‐9‐CM)/International Classification of Diseases, Tenth Revision, Clinical Modification (ICD‐10‐CM) diagnosis codes (Table S1) were used to identify any and each specific type of irAE by scanning the primary or secondary diagnoses on inpatient claims (Medicare Part A) and physician visit claims (Medicare Part B). The primary endpoint of this study was the occurrence of incident irAEs (any or specific type), and the secondary endpoint was the timing of irAE incidence after ICI initiation.

### 2.4 Risk factor variables

Risk factors for irAE incidence evaluated in this study included patient demographics, cancer‐related characteristics, and clinical history. Patients' demographic information included age, sex, race, ethnicity, marital status, geographic region, urban residence, census tract poverty status, and dual Medicare and Medicaid eligibility. Cancer‐related risk factors included time from first NSCLC diagnosis to ICI initiation, cancer stage at the time of diagnosis, tumor histology at diagnosis, and the presence of central nervous system (CNS) metastasis (identified in the medical claims based on diagnosis codes) in a 6‐month period before ICI initiation. Cancer treatment history was also identified, including NSCLC‐related surgery and radiation therapy from NSCLC diagnosis to ICI initiation. Adjuvant chemotherapy with index ICI treatment was identified if received within the 28 days (±14 days of index date) of the first administered ICI. The treatment line of ICI regimens was determined based on a published algorithm by Hess et al.^27^ In addition, other important clinical history variables, such as any history of autoimmune disorders, the NCI Comorbidity Index,^28^ and disability status,^29^, ^30^ were captured from the medical claims within 12 months before the initiation of ICIs. Lastly, recent use of corticosteroids was measured in the 3‐month period prior to the index date.

### 2.5 Statistical analysis

Descriptive statistics were used to depict baseline patient characteristics and irAE characteristics. *t*‐Tests or Mann–Whitney *U* tests were used to assess differences in continuous baseline variables between patients with irAEs and patients without irAEs, as appropriate. Chi‐squared tests were used to assess differences among categorical variables between the two groups, as appropriate. Given death or initiation of a subsequent line of treatment may occur before patients develop an irAE, the occurrence of these events would preclude the observation of the irAEs. The competing risk of these events might bias the estimation of predictive effect of covariates in a conventional survival analysis. Therefore, the Fine–Gray regression model^31^ was used to assess the association between risk factors and the time to develop the first occurrence of irAEs (any type) adjusting for competing risk of all‐cause death and advancing to subsequent line of therapy in the cohort of patients with NSCLC who received ICI treatment. The subsequent line of therapy post‐ICI‐containing regimens was identified if patients started single‐agent chemotherapy (e.g., pemetrexed, docetaxel, or gemcitabine), other ICI agents, or docetaxel plus ramucirumab. To further analyze the risk factors associated with first occurrence of a specific type of irAEs, separate multivariable models were estimated for select types of irAEs, including pneumonitis, hypothyroidism, arrhythmia, and acute kidney injury (AKI). All analyses were performed using SAS version 9.4 (SAS Institute).

## 3 RESULTS

**FIGURE 1:**
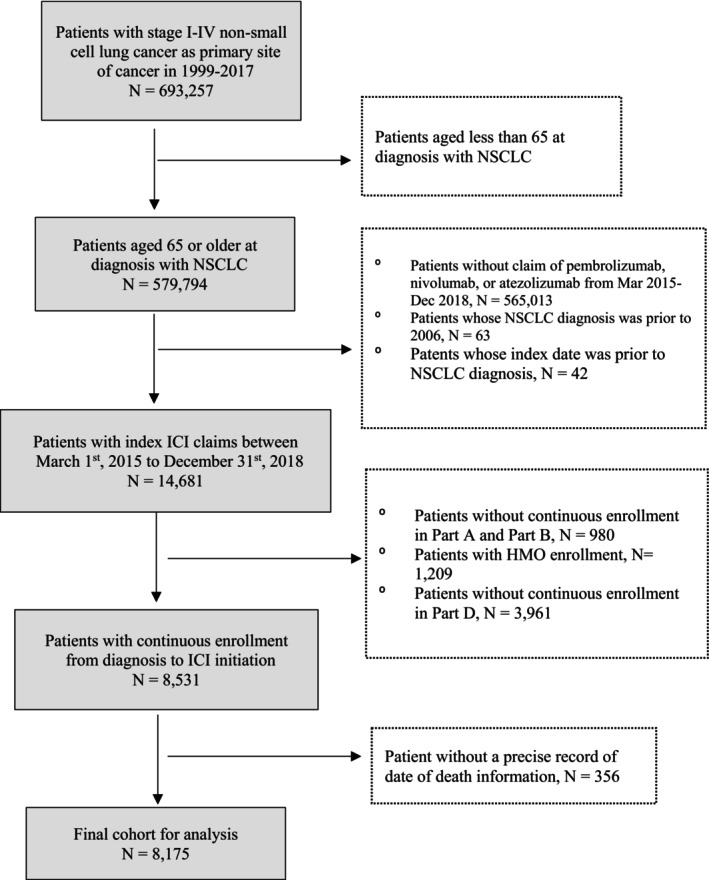
Cohort selection diagram.

In total, 8175 older patients with NSCLC who underwent ICI treatment were included in the study (Figure 1), averaging 75 (±6.03) years of age (Table 1). Among them, 88.0% were White and 49.0% were male. A majority of patients (86.6%) lived in metropolitan areas, over 40% came from the registries in Northeast (40.7%), and almost half (43.3%) were married or had a partner. Adenocarcinoma and squamous subtypes comprised 58.1% and 28.0% of patients, respectively. At diagnosis, more than half (51.6%) the patients had metastatic disease. Mean time from NSCLC diagnosis to index ICI initiation was 16.57 (±18.42) months. A majority (59.5%) of patients initiated nivolumab at index and 49.2% of patients started ICI as the second‐line treatment. Before ICI initiation, the majority of patients received radiation therapy (59.2%), were on corticosteroids (63.9%), and more than half (52.4%) had “better” disability status.

**TABLE 1:** Patient characteristics and irAE incidence.

Variable	Total sample (*N* = 8175)		IrAEs group (*N* = 3826)		No irAEs group (*N* = 4349)		*p* ^a^
	Mean/*N*	SD/%	Mean/*N*	SD/%	Mean/*N*	SD/%	
Demographic characteristics^b^							
Age at ICI initiation (mean)	75.56	6.03	75.73	6.04	75.41	6.01	0.01
Age categories^c^							0.15
65–74	3871	47.4%	1768	45.7%	2103	54.3%	
75–84	3567	43.6%	1705	47.8%	1862	52.2%	
85 years+	737	9.0%	353	47.9%	384	52.1%	
Sex							0.23
Male	4008	49.0%	1903	47.5%	2105	52.5%	
Female	4167	51.0%	1923	46.2%	2244	53.9%	
Race							0.87
White	7193	88.0%	3373	46.9%	3820	53.1%	
Black	531	6.5%	242	45.6%	289	54.4%	
Others/unknown	451	5.5%	211	46.8%	238	52.8%	
Ethnicity (Spanish–Hispanic–Latino)	364	4.5%	162	44.5%	202	55.5%	0.37
Marital status							0.12
Live independently	2410	29.5%	1117	46.4%	1293	53.7%	
Live with spouse or partner	3543	43.3%	1628	46.0%	1915	54.1%	
Unknown	2222	27.2%	1081	48.7%	1141	51.4%	
Socioeconomic status							
Dual enrolled to Medicaid and Medicare at baseline	1449	17.7%	686	47.3%	763	52.7%	0.65
Census tract poverty							0.03
0%–5% poverty	1996	24.4%	960	48.1%	1036	51.9%	
5%–10% poverty	2059	25.2%	992	48.2%	1067	51.8%	
10%–20% poverty	2106	25.8%	956	45.4%	1150	54.6%	
20%–100% poverty	1186	14.5%	515	43.4%	671	56.6%	
Unknown	828	10.1%	403	48.7%	425	51.3%	
Metropolitan residence	7077	86.6%	3338	47.2%	3739	52.8%	0.09
Geographic region							<0.001
West	2678	32.8%	1167	43.6%	1511	56.4%	
Northeast	3326	40.7%	1671	50.2%	1655	49.8%	
Midwest	652	8.0%	320	49.1%	332	50.9%	
South	1519	18.6%	668	44.0%	851	56.0%	
Cancer‐related characteristics							
Months from diagnosis to ICI initiation	16.6	18.4	16.6	18.4	16.6	18.5	0.32
Stage							0.40
Nonmetastatic	3790	46.4%	1789	47.2%	2001	52.8%	
Metastatic	4214	51.5%	1950	46.3%	2264	53.7%	
Unknown	171	2.1%	87	50.9%	84	49.1%	
Histology							0.33
Adenocarcinoma	4747	58.1%	2242	47.2%	2505	52.8%	
Squamous cell	2286	28.0%	1058	46.3%	1228	53.7%	
Large cell	85	1.0%	32	37.7%	53	62.4%	
Other/ not otherwise specified	1057	12.9%	494	46.7%	563	53.3%	
ICI‐related characteristics							
ICI regimens							<0.001
Atezolizumab	311	3.8%	115	37.0%	196	63.0%	
Nivolumab	4861	59.5%	2158	44.4%	2703	55.6%	
Pembrolizumab	3003	36.7%	1553	51.7%	1450	48.3%	
Chemotherapy combined with ICI							<0.001
In combination	860	10.5%	452	52.6%	408	47.4%	
ICI monotherapy	7315	89.5%	3374	46.1%	3941	53.9%	
Therapy line							<0.001
1L	2411	29.5%	1228	50.9%	1183	49.1%	
2L	4026	49.2%	1851	46.0%	2175	54.0%	
3L+	1738	21.3%	747	43.0%	991	57.0%	
ICI treatment duration (month)	6.6	8.5	8.9	9.9	4.6	6.4	<0.001
Cancer‐related therapies history							
Radiation therapy before ICI start	4841	59.2%	2226	46.0%	2615	54.0%	0.07
Cancer‐directed surgery before ICI start	1760	21.5%	845	48.0%	915	52.0%	0.25
Clinical history							
NCI comorbidity index	0.54	0.46	0.55	0.47	0.53	0.46	0.03
CNS metastasis in 6 months before ICI initiation	1382	16.9%	574	41.5%	808	58.5%	<0.001
Disability status							0.22
No	4287	52.4%	2034	47.5%	2253	52.6%	
Yes	3888	47.6%	1792	46.1%	2096	53.9%	
Autoimmune disorder before ICI initiation	1640	20.1%	811	49.5%	829	50.6%	0.02
Any use of corticosteroids before 90 days of ICI initiation	5158	63.1%	2382	46.2%	2776	53.8%	0.14

Abbreviations: 1L, first line; 2L, second line; 3L, third line; ICI, immune checkpoint inhibitors; SD, standard deviation.

^a^ *p*‐value for univariable analyses for assessing the association between the given characteristic and whether or not an irAE was experienced.

^b^ For categorical characteristics, for the columns labeled “IrAEs group” and “No irAEs group,” the percents are row percentages and thus represent what percentage of each category experienced or did not experience an irAE, respectively.

^c^ Age at ICI initiation.

A total of 3826 (46.8%) patients developed an irAE after treatment initiation. A comparison between the demographic and clinical characteristics of both the irAE and non‐irAE cohorts is provided in Table 1. Higher incidence of irAEs was observed among patients on first‐line ICI treatment at index (50.9% vs. second‐line: 46.0% and third‐line or later: 43.0%, *p* < 0.001), receiving pembrolizumab (51.7% vs. nivolumab: 44.4% and atezolizumab: 37.0%, *p* < 0.001), and receiving ICI treatment combined with chemotherapy (52.6% vs. 46.1%, *p* < 0.001). The incidence of irAEs was higher for patients who had autoimmune disorders before ICI initiation (49.5%, vs. 46.1%, *p* = 0.02) and lower for patients who had CNS metastasis at NSCLC diagnosis (41.5% vs. 47.9%, *p* < 0.001). In addition, those who experienced irAEs had a longer ICI treatment duration than those who did not (8.9 months vs. 4.6 months, *p* < 0.001) and had a higher NCI score (0.55 vs. 0.53, *p* = 0.03).

With regard to organ systems affected by the irAE event, 16.5% of patients developed pulmonary irAEs post‐ICI initiation, followed by cardiac (15.0%), endocrine (12.9%), renal (6.1%), dermatological (5.3%), hematologic (5.1%), GI (3.5%), hepatic (hepatitis, 2.1%), musculoskeletal (1.7%), and CNS (1.1%) irAEs. The most common irAEs across all organ systems were pneumonitis (16.5%), arrhythmia (11.2%), hypothyroidism (10.4%), and AKI (5.8%). Incidence of some rare irAEs, such as colitis (2.8%), hepatitis (2.1%), type 1 diabetes (1.2%), adrenal insufficiency (1.1%), cardiomyopathy (1.1%), myocarditis (1.1%), and hypophysitis (0.2%), was also observed among our study sample (see Tables S2 and S3 for further breakdown).

The median time to irAE occurrence was 82 days (IQR: 29–182 days). Among the patients who experienced an irAE, 53.3% developed their first irAE within 3 months of starting ICI, and 25.1% developed them over 6 months. The earliest onset of irAE occurrence was for hematologic irAEs (median = 75 days, IQR: 31.5–171.5 days), whereas the latest was musculoskeletal (median = 177, IQR: 86–324 days) irAEs (Table S4).

**FIGURE 2:**
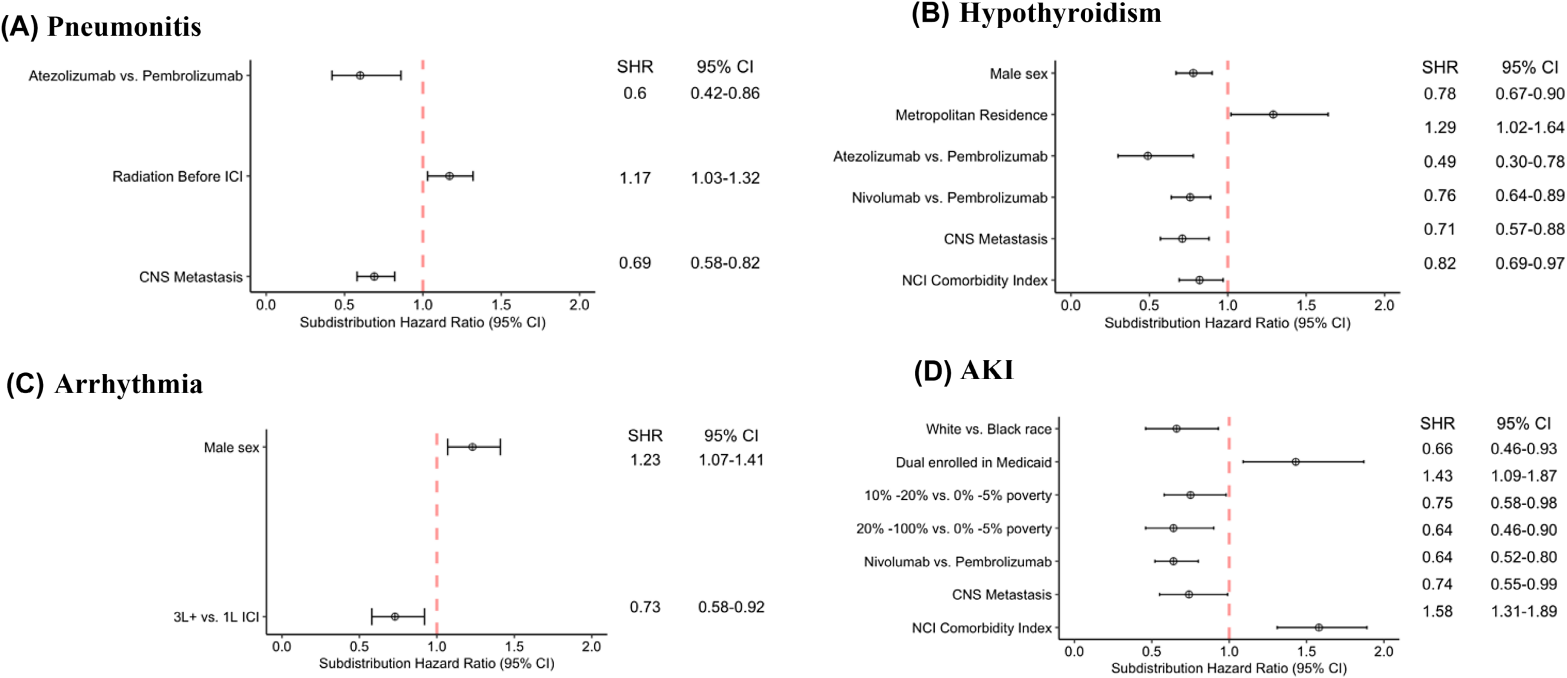
Significant risk factors for predicting the incidence of specific irAEs: (A) pneumonitis, (B) hypothyroidism, (C) arrhythmia, and (D) AKI

The risk factors for the first occurrence of any irAEs were evaluated using the Fine–Gray model (Table 2). After excluding patients with missing values in the variables of interest, 7999 patients were retained for the analysis (i.e., around 2% of cases had any missing data). Immune checkpoint inhibitor type (atezolizumab vs. pembrolizumab [subdistribution hazard ratio (SHR): 0.72, 95% CI: 0.59–0.88]; nivolumab vs. pembrolizumab [SHR: 0.88, 95% CI: 0.81–0.95]), combination of chemotherapy with ICI vs. Immune checkpoint inhibitor monotherapy (SHR: 1.15, 95% CI: 1.03–1.28), therapeutic line of ICI (third line or later vs. first line [SHR: 0.85, 95% CI: 0.77–0.95]), CNS metastasis (SHR: 0.82, 95% CI: 0.75–0.91), history of autoimmune disorder (SHR: 1.10, 95% CI: 1.02–1.20), and NCI comorbidity score (SHR: 1.08, 95% CI: 1.00–1.16) were identified as significant predictors. The significant risk factors of incident pneumonitis, hypothyroidism, arrythmia, and AKI were also assessed using the Fine–Gray model and are presented in the Figure 2A–D (for full results, see Tables S5–S8).

**TABLE 2:** Fine–Gray model predicting the incidence of any irAEs.

Risk factor		Any IrAEs			
		SHR	95% CI		*p*
Age	65–74 (ref)				
	75–84	1.06	0.99	1.13	0.10
	85 years+	1.04	0.92	1.17	0.55
Sex	Female (ref)				
	Male	1.05	0.99	1.13	0.13
Race	Black (ref)				
	White	0.98	0.85	1.13	0.76
	Other	1.02	0.84	1.25	0.83
Hispanic	Yes vs. No	0.94	0.80	1.11	0.48
Region	West (ref)				
	Midwest	1.19	1.05	1.35	0.007
	Northeast	1.24	1.13	1.36	<0.001
	South	0.98	0.88	1.09	0.70
Marital status	Live independently (ref)				
	Live with spouse or partner	0.99	0.91	1.08	0.84
	Unknown	0.94	0.85	1.04	0.19
Dual enrolled	Yes vs. No	1.07	0.98	1.18	0.13
Census tract poverty	0%–5% poverty (ref)				
	5%–10% poverty	1.06	0.97	1.16	0.24
	10%–20% poverty	1.00	0.91	1.10	0.97
	20%–100% poverty	0.94	0.83	1.06	0.29
	Unknown	1.05	0.92	1.20	0.48
Metropolitan residence	Yes vs. No	1.05	0.95	1.16	0.39
ICI	Pembrolizumab (ref)				
	Atezolizumab	0.72	0.59	0.88	0.001
	Nivolumab	0.88	0.81	0.95	0.001
Chemotherapy combined with ICI	In combination with chemotherapy vs. ICI monotherapy	1.15	1.03	1.28	0.01
Months from diagnosis to ICI initiation		1.00	0.99	1.00	0.30
Cancer stage	Metastatic (ref)				
	Not metastatic	0.99	0.92	1.07	0.79
Histology	Adenocarcinoma (ref)				
	Large cell	0.82	0.58	1.17	0.27
	Other/not otherwise specified	0.99	0.90	1.09	0.85
	Squamous cell	0.99	0.92	1.07	0.82
Line of therapy For ICI	1L (ref)				
	2L	0.92	0.85	1.01	0.07
	3L+	0.85	0.77	0.95	0.01
Radiation before ICI	Yes vs. No	1.03	0.96	1.11	0.45
Surgery before ICI	Yes vs. No	1.04	0.95	1.13	0.43
CNS metastasis	Yes vs. No	0.82	0.75	0.91	<0.001
Autoimmune disorder	Yes vs. No	1.10	1.02	1.20	0.02
Disability status	Yes vs. No	0.99	0.93	1.06	0.78
Recent use of steroids	Yes vs. No	1.02	0.95	1.09	0.60
NCI comorbidity index		1.08	1.00	1.16	0.049

Abbreviations: 1L, first line; 2L, second line; 3L, third line; CI, confidence interval; ICI, immune checkpoint inhibitors; irAE, immune‐related adverse event; SHR, subdistribution hazard ratio.

## 4 DISCUSSION

In our study, nearly half of the older patients with NSCLC experienced at least one irAE after ICI initiation. Pneumonitis, arrhythmia, hypothyroidism, and AKI were among the most common irAEs. The median interval from ICI initiation to irAE onset was found to be around 3 months, but one‐fourth of patients developed their first irAE after 6 months. After accounting for competing risks of death and treatment discontinuation, greater hazards of irAE incidence were particularly associated with patients who received certain type of ICI at index, did not have CNS metastases, had a history of autoimmune disorder, and had chemotherapy in combination with ICI for older adults with NSCLC. Unique risk factors were also found specifically be associated with the occurrence of certain types of irAEs. To the best of our knowledge, this is the first study to investigate the incidence and risk factors of irAE incidence among older Medicare beneficiaries who have been diagnosed with NSCLC and initiated treatment with ICIs.

Few studies have investigated the incidence of irAEs after ICI initiation among older patients with NSCLC in a real‐world setting. A study by Muchnik et al. conducted on 75 older patients with NSCLC from one academic hospital found that 37% experienced irAEs of any grade.^7^ Pneumonitis and thyroiditis were the most common irAEs, followed by colitis and dermatitis.^7^ Our findings are consistent with this spectrum, although we observe a higher occurrence of incident irAEs in the current study. This may be due to the discrepancy in study design and data source. Although the limitations of the database used for the current study prevent robust direct comparisons between older and younger adults, it is worth noting that the incidence of any irAEs in our study was similar to that reported in a large retrospective study of younger NSCLC patients.^16^ Some studies have suggested that irAEs occur with no significantly different incidence between older and younger adults.^32^, ^33^, ^34^ Older NSCLC patients in our study, however, were observed to have a higher incidence of pneumonitis, hypothyroidism, AKI, and arrhythmia compared with the study evaluating younger patients.^16^ This suggests unique characteristics of the incidence irAEs among older patients that may be attributed to the impairment of their immune systems or dysfunction in particular organs. It is important to monitor such events and provide early intervention during ICI treatment. In addition, our study observed a low incidence (~1%) of some irAEs, such as adrenal insufficiency, type 1 diabetes, hypophysitis, cardiomyopathy, and myocarditis, comparable to other observational studies in patients of mixed ages and tumor types, suggesting that these irAEs are similarly rare among older patients with NSCLC. These events may, however, be serious and fatal; further research is necessary to assess their severity and impact on older patients.^16^, ^35^, ^36^

Sparse data are available to describe the timing of irAEs in older adults with NSCLC. Based on our study, the median time to occurrence of any type of irAEs was 82 days after ICI initiation at the median, and pneumonitis—the most common irAE—occurred 106 days after ICI initiation. This is consistent with Daniello et al.'s findings in German NSCLC patients at stage IV of any age, in which the median irAE time‐to‐onset from ICI start was 3.1 months, with 105 days for lung irAEs.^17^ Nevertheless, another real‐world study, which evaluated patients with various cancers, including NSCLC, and reported a shorter time to onset (median: 9.8 weeks).^37^ This indicates that older patients with NSCLC may experience a relatively late onset of irAEs compared to patients with other cancers. We found that irAE incidence increased cumulatively up to 12 months and occurred even after treatment discontinuation, which is in line with the results of other retrospective studies.^16^ It has been reported that patients in advanced stage of NSCLC had a short time to onset of heart and liver irAEs and a longer onset of endocrine and kidney irAEs.^17^ Contrary to these findings, our study observed that the cardiovascular and liver irAEs developed later, but endocrine and renal toxicities were present earlier in our study cohort. This may be due to our study was conducted in a patient population with specific baseline characteristic and from a difference healthcare system. Given the fact that around half of our study sample were diagnosed with NSCLC at the nonmetastatic stage, these findings may indicate that older patients and/or patients at an earlier stage of the disease may experience a different pattern of irAEs onset.

The significant risk factors identified in our study, such as sex, comorbidity burden, and first‐line ICI, were consistent with what previous research have examining in general NSCLC population.^17^, ^38^, ^39^, ^40^, ^41^, ^42^ Meanwhile, we offer evidence in areas where there is controversy in the literature, including whether autoimmune disorders (AID), radiation therapy, and chemotherapy all contribute to irAEs development. A history of AID was previously reported to be associated with an increased flare‐up of AID or an increased risk for irAEs.^43^ Yet, in a different study of NSCLC patients, there was no significant association between baseline AID and either the development, grade, or time to irAE.^44^ An analysis of retrospective data in Mexico showed that patients with radiation therapy treated with second‐line immunotherapy had a significantly higher incidence of pneumonitis. There are, however, some studies that have not found a significant correlation between prior thoracic radiation therapy and pneumonitis.^45^, ^46^, ^47^ In Daniello et al.'s study, no significant difference was found between chemoimmunotherapy and mono‐ICI therapy.^17^ In contrast to this, a meta‐analysis of RCTs in NSCLC demonstrated that the rate of most irAEs could be reduced by combining ICI with chemotherapy.^48^ This may reflect that the impact of autoimmune diseases, prior cancer treatment, and immunochemotherapy can be detrimental to older patients who are eligible for immunotherapy.

In addition, we identified a few distinctive features, which have been rarely evaluated in the literature, of predicting the occurrence of specific type of irAEs, of which the incidence was found to be higher in older NSCLC patients. First, in our cohort, the presence of CNS metastases is linked to a lower risk of irAEs, especially pneumonitis, hypothyroidism, and AKI, which are the most common irAEs in our cohort. Randomized controlled trials typically exclude patients with active CNS metastases. Research suggests that ICIs can benefit NSCLC patients who have brain metastases with acceptable toxicity.^49^ Studies exploring mechanisms are needed for further prospective validation. Second, our study suggests that pembrolizumab may also increase the risk of irAEs, including pneumonitis, hypothyroidism, and AKI, as compared to other agents. The choice of a treatment plan for older patients should be made with caution. Furthermore, unlike other irAEs, hypothyroidism occurs more frequently among patients with a low NCI index score and at early cancer stages. Since the incidence of hypothyroidism has been found to be associated with survival benefit,^41^, ^50^ our study provided evidence in the hypothesis that the occurrence of hypothyroidism may reflect a response to ICI. Our study observed that AKI was much more prevalent than what was reported in RCTs; however, the incidence and risk factor of it was less investigated in the current literature.^13^, ^51^, ^52^ We also discovered that irAE rates vary based on patients' socioeconomic status. Black patients, dual‐enrolled Medicaid individuals, and patients residing in nonmetropolitan areas had a higher risk of developing irAEs, especially AKI. There may be a connection between low socioeconomic status of these patient populations and their vulnerability in terms of their health and less accessibility to oncologists and medicine subspecialists.

The results of this study must be viewed in the context of certain limitations. First, although patients were well‐characterized in demographic characteristics and NSCLC histology variables, SEER‐Medicare data do not provide information regarding PD‐L1 expression level, smoking status, body mass index (BMI), or the status of other potential biomarkers. Therefore, the association between these variables and the incidence of irAEs cannot be evaluated in the current study. According to recent studies, patients' human leukocyte antigen (HLA) expression may be predictive of their risk and severity of irAEs. Future studies should incorporate genomic test data to assess the association between HLA status and the development of irAE for older patients.^53^, ^54^, ^55^ There is also a lack of data about irAE severity (e.g., Common Terminology Criteria for Adverse Events grading) in the SEER‐Medicare linked database. It is also necessary to conduct future studies to assess the incidence and risk factors associated with severe irAEs. Furthermore, some variables in the SEER Cancer file could not be adequately analyzed due to missing information, especially from three registries. Second, the analysis was limited to NSCLC patients enrolled in fee‐for‐service Medicare. Consequently, findings from this study are not generalizable to older adults with NSCLC enrolled in HMOs (also known as Medicare Advantage). Third, relying on ICD‐9‐CM/ICD‐10‐CM diagnosis codes and CPT/HCPSC procedure codes may lead to misclassification bias, underestimating irAEs, ICI use, and risk factor classification (e.g., the identification of CNS metastasis based on ICD diagnosis codes might be incomplete or inaccurate).

## 5 CONCLUSION

This real‐world study highlighted the significant high incidence and unique spectrum of irAEs in older patients with NSCLC. Race, socioeconomic status, cancer status, treatment variables, and comorbidity burden were specifically associated with increased risk of a certain type of irAEs. Accordingly, it is imperative that older adults with NSCLC be closely monitored and the occurrence of irAEs should be anticipated.

## AUTHOR CONTRIBUTIONS

**Yiran Rong:** Conceptualization (lead); formal analysis (lead); investigation (lead); methodology (lead); software (lead); writing – original draft (lead). **John P. Bentley:** Conceptualization (equal); formal analysis (supporting); methodology (equal); supervision (equal); writing – review and editing (equal). **Kaustuv Bhattacharya:** Writing – review and editing (equal). **Yi Yang:** Writing – review and editing (equal). **Yunhee Chang:** Writing – review and editing (equal). **Sally Earl:** Writing – review and editing (equal). **Sujith Ramachandran:** Conceptualization (equal); formal analysis (supporting); methodology (equal); supervision (equal); writing – review and editing (equal).

## FUNDING INFORMATION

None.

## CONFLICT OF INTEREST STATEMENT

None.

## ETHICS STATEMENT

This study was approved by the Institutional Review Board at the University of Mississippi (protocol #21–036).

## Supporting information


Table S1

## Data Availability

The data for this study were obtained through a data use agreement with the National Cancer Institute and cannot be shared publicly. Data can be accessed, subject to approval and data use agreement, from the Healthcare Delivery Research Program at the National Cancer Institute (http://appliedresearch.cancer.gov/seermedicare/obtain/requests.html).
