# Supplementary material for: Incidence and risk factors of immune‐related adverse events induced by immune checkpoint inhibitors among older adults with non‐small cell lung cancer
Source: Cancer Med. 2024 Jan 2;13(1):e6879. doi: 10.1002/cam4.6879 (PMC10807682; doi:10.1002/cam4.6879)
Supplement: Supplementary file 1 — Table S1 [file CAM4-13-e6879-s001.docx]

**Supporting Information**

**Table S1 Immune-related adverse events of interest and ICD-9-CM/ICD-10-CM codes**

| **Organ system** | **Category** | **ICD-9 codes** | **ICD-10 codes** |
| --- | --- | --- | --- |
| **Dermatologic** | Eruption | 693.0 | L27.0, L27.1 |
|  | Pruritus | 698.8 | L29.8, L29.9 |
|  | Lichen planus | 697.0 | L43.x, L44.3, L66.1 |
|  | Vitiligo | 709.00, 709.01, 709.09 | L80, L81.8, L81.9 |
|  | Bullous Pemphigoid | 694.5 | L12.0 |
|  | Stevens-Johnson syndrome (SJS)/ toxic epidermal necrolysis (TEN) | 695.13, 695.14 | L51.1, L51.3 |
| **Hematologic** | Anemia | 285.3, 285.8, 285.9284.x, 283.x | D59.x, D61.x, D60.x, D64.2, D64.3, D64.8 |
|  | Thrombocytopenia | 287.3, 287.31, 287.8, 287.9, 287.31, 287.32, 287.4,  287.49, 287.5287.8, 287.9 | D69.3, D69.41, D69.49, D69.59, D69.6 |
|  | Leukopenia | 288.00, 288.03, 288.09 288.4, 288.5x, 288.8, 288.9 | D72.1, D72.81, D72.810, D72.818, D72.819, D70.9,  D70.4, D70.2, D76.1, D76.3 |
| **Pulmonary** | Pneumonitis | 508.8, 508.9, 486.x, 516.3x, 516.9 | J84.11x, J70.9, J70.8, J70.2, J70.3, J70.4, J18.9,  J84.89, J84.9 |
| **Liver** | Hepatitis | 573.3, 790.5 | K71, R74.8, K75.4, K75.9, R94.5 |
| **Gastrointestinal** | Colitis | 558.2, 558.3, 558.4, 558.9, 555, 556.8, 556.9 | K52.1, K52.29, K52.3, K52.9, K52.89, K53.82 |
|  | Pancreatitis | 577.0 | K85.x |
|  | Mucositis | 528.x | K12.30, K12.31 |
| **Endocrine** | Hypothyroidism | 244.3, 244.8, 244.9 | E03.2, E03.8, E03.9 |
|  | Hyperthyroidism | 242.x | E05.x |
|  | Diabetes Type I | 250.01, 250.03, 250.11, 250.13, 250.21, 250.23, 250.31, 250.33, 250.41, 250.43, 250.61, 250.63, 250.71, 250.73, 250.81, 250.83, 250.91, 250.93 | E09.x, E10.x, E13 |
|  | Diabetic ketoacidosis (DKA) | 250.11, 250.13 | E10.1 |
|  | Hypophysitis | 253.8 | E23.6 |
|  | Adrenal insufficiency | 255.41 | E27.1, E27.3, E27.4 |
| **Renal** | Acute Kidney Injury (AKI) | 584.x | N17.x |
|  | Acute interstitial nephritis | 591.10, 591.11, 583.89 | N10.x, N12.x, N14.1, N14.2 |
|  | Glomerulonephritis | 580.x, 581.x, 583.x | N00.x, N01.x, N04.x, N05.x N06.x, |
| **Cardiac** | Arrhythmia | 427.x | I48.x, I47.x, I49.x, I46.x |
|  | Acute MI | 410.x | I21.x |
|  | Myocarditis | 422.x, 429.x | I40.x, I51.4, I51.8, I51.9 |
|  | Pericarditis | 420.x, 423.x | I30.x I31.4, I31.8-9 |
|  | Cardiomyopathy | 425.4, 425.9 | I42.0, I42.7, I42.9 |
| **Musculoskeletal** | Dermatopolymyositis/ polymyositis | 710.3, 710.4 | M33 |
|  | Rheumatoid arthritis | 714.x | M05, M06, M08.0, M08.2, M08.3, M08.4, M08.8, M08.9, M12.0 |
|  | Myalgia | 729.1 | M79.1 |
|  | Polymyalgia rheumatica (PMR) and giant cell arteritis (GCA) | 725, 446.5 | M35.5, M31.5, M31.6 |
|  | Neuritis/neuralgia | 729.2 | M79.2 |
| **Central Nervous** **System (CNS)** | Meningitis | 047.9, 322.9 | G03.9, A87.9 |
|  | Encephalitis/myelitis/ encephalomyelitis | 323.8, 323.81, 323.82, 323.7, 323.71, 323.72, 323.9 | G92, G04.81, G04.89, G04.90, G04.91 |
|  | Guillain-Barre syndrome (GBS) | 357.0 | G61.0 |
|  | Myasthenia gravis | 358.0 | G70.0 |
|  | Peripheral neuropathy | 357.89 | G64 |

**Table S2 IrAE incidence by organ system/category (N=3,826)**

|  | **Overall** | |
| --- | --- | --- |
| **Organ system/ Category** | **N** | **%** |
| **Any** | 3,826 | 46.8 |
| **Pulmonary** (Pneumonitis) | 1,350 | 16.5 |
| **Cardiac** | 1,228 | 15.0 |
| Arrhythmia | 914 | 11.2 |
| Acute MI | 210 | 2.6 |
| Cardiomyopathy | 89 | 1.1 |
| Myocarditis | 92 | 1.1 |
| Pericarditis | 67 | 0.8 |
| **Endocrine** | 1,054 | 12.9 |
| Hypothyroidism | 854 | 10.5 |
| Hyperthyroidism | 111 | 1.4 |
| Adrenal insufficiency | 88 | 1.1 |
| Diabetes Type I/Diabetic ketoacidosis (DKA) | 95 | 1.2 |
| Hypophysitis | 13 | 0.2 |
| **Renal** | 501 | 6.1 |
| Acute Kidney Injury (AKI) | 474 | 5.8 |
| Acute interstitial nephritis/ Glomerulonephritis | 44 | 0.5 |
| **Dermatologic** | 436 | 5.3 |
| Eruption/Rash | 313 | 3.8 |
| Pruritus | 113 | 1.8 |
| Lichen planus | 38 | 0.5 |
| Vitiligo | 14 | 0.2 |
| Bullous Pemphigoid/ Stevens-Johnson syndrome (SJS)/ toxic epidermal necrolysis (TEN) | 14 | 0.2 |
| **Hematologic** | 420 | 5.1 |
| Anemia | 264 | 3.2 |
| Leukopenia | 88 | 1.1 |
| Thrombocytopenia | 105 | 1.3 |
| **Gastrointestinal** | 285 | 3.5 |
| Colitis | 226 | 2.8 |
| Pancreatitis | 39 | 0.5 |
| Mucositis | 24 | 0.3 |
| **Liver** (Hepatitis) | 170 | 2.1 |
| **Musculoskeletal** | 141 | 1.7 |
| Dermatopolymyositis/ polymyositis/Polymyalgia rheumatica (PMR) and giant cell arteritis (GCA) | 29 | 0.4 |
| Rheumatoid arthritis | 46 | 0.6 |
| Myalgia | 75 | 0.9 |
| **CNS** | 89 | 1.1 |
| Encephalitis/myelitis/ encephalomyelitis/Myasthenia gravis | 53 | 0.6 |
| Neuritis/neuralgia/Meningitis/ Guillain-Barre syndrome (GBS) /Peripheral neuropathy | 38 | 0.4 |

**Table S3 IrAE incidence by organ system/category (N=3,826) stratified by age**

| **Organ system/ Category** | **65-74** | | **75-84** | | **85 years +** | |
| --- | --- | --- | --- | --- | --- | --- |
|  | **N** | **%** | **N** | **%** | **N** | **%** |
| **Any** | 1768 | 45.7% | 1705 | 47.8% | 353 | 47.9% |
| **Pulmonary** (Pneumonitis) | 614 | 15.9% | 600 | 16.8% | 136 | 18.5% |
| **Cardiac** | 550 | 14.2% | 558 | 15.6% | 120 | 16.3% |
| Arrhythmia | 399 | 10.3% | 422 | 11.8% | 93 | 12.6% |
| Other cardiac irAEs | 219 | 5.7% | 205 | 5.7% | 34 | 4.6% |
| **Endocrine** | 484 | 12.5% | 482 | 13.5% | 88 | 11.9% |
| Hypothyroidism | 385 | 9.9% | 389 | 10.9% | 80 | 10.9% |
| Other endocrine irAEs | 155 | 4.0% | 139 | 3.9% | 13 | 1.8% |
| **Renal** | 216 | 5.6% | 231 | 6.5% | 54 | 7.3% |
| Acute Kidney Injury (AKI) | 204 | 5.3% | 219 | 6.1% | 51 | 6.9% |
| **Dermatologic** | 185 | 4.8% | 211 | 5.9% | 40 | 5.4% |
| Eruption/Rash | 127 | 3.3% | 157 | 4.4% | 29 | 3.9% |
| Other dermatologic irAEs | 85 | 2.2% | 77 | 2.2% | 17 | 2.3% |
| **Hematologic** | 197 | 5.1% | 197 | 5.5% | 26 | 3.5% |
| Anemia | 125 | 3.2% | 121 | 3.4% | 18 | 2.4% |
| **Gastrointestinal** | 148 | 3.8% | 117 | 3.3% | 20 | 2.7% |
| Colitis | 122 | 3.2% | 86 | 2.4% | 18 | 2.4% |
| **Liver** (Hepatitis) | 79 | 2.0% | 79 | 2.2% | 12 | 1.6% |

Note: In accordance with the SEER-Medicare Data Use Agreement (DUA), cells with patient count less than 11 were collapsed with other AE categories or not reported in this table.

**Table S4 Distribution of Patients with irAEs by Onset Time**

| **Organ system/ Category** | **N** | **Less than 3 months** | | **3-6 months** | | **Over 6 months** | |
| --- | --- | --- | --- | --- | --- | --- | --- |
|  |  | **N** | **%** | **N** | **%** | **N** | **%** |
| **Any** | 3,826 | 2,039 | 53.3 | 826 | 21.6 | 961 | 25.1 |
| **Hematologic** | 420 | 233 | 55.5 | 87 | 20.7 | 100 | 23.8 |
| **CNS**^†^ | 89 | 60 | 67.4 | - | - | 229 | 32.6 |
| **Cardiac** | 1,228 | 568 | 46.3 | 244 | 19.9 | 416 | 33.9 |
| **Pulmonary** | 1,350 | 604 | 44.7 | 292 | 21.6 | 454 | 33.6 |
| **Renal** | 501 | 222 | 44.3 | 113 | 22.6 | 166 | 33.1 |
| **Endocrine** | 1,054 | 467 | 44.3 | 246 | 23.3 | 341 | 32.4 |
| **Hepatic** | 170 | 68 | 40.0 | 34 | 20.0 | 68 | 40.0 |
| **Gastrointestinal** | 285 | 94 | 33.0 | 64 | 22.5 | 127 | 44.6 |
| **Dermatologic** | 436 | 132 | 30.3 | 99 | 22.7 | 205 | 47.0 |
| **Musculoskeletal** | 141 | 39 | 27.7 | 32 | 22.7 | 70 | 49.7 |

† Due to few patients had incident CNS irAEs occurred from 3- to 6-month post index, those patients were collapsed with those who had irAEs less than 3 months.

**Table S5 Fine-Gray model for predicting the incidence of pneumonitis**

| **Risk factor** | | **Pneumonitis** | | | |
| --- | --- | --- | --- | --- | --- |
|  |  | **SHR** | **95% CI** | | ***p*** |
| **Age** |  | 1.01 | 0.99 | 1.02 | 0.19 |
| **Sex** | Female (ref) |  |  |  |  |
|  | Male | 1.11 | 0.99 | 1.25 | 0.07 |
| **Race** | Black (ref) |  |  |  |  |
|  | White | 0.99 | 0.78 | 1.25 | 0.92 |
|  | Other | 1.16 | 0.83 | 1.62 | 0.38 |
| **Hispanic** | Yes vs No | 1.17 | 0.91 | 1.51 | 0.23 |
| **Region** | West (ref) |  |  |  |  |
|  | Midwest | 1.23 | 1.00 | 1.52 | 0.05 |
|  | Northeast | 1.04 | 0.88 | 1.22 | 0.66 |
|  | South | 1.00 | 0.83 | 1.20 | 0.99 |
| **Marital Status** | Live independently (ref) |  |  |  |  |
|  | Live with souse or partner | 0.92 | 0.80 | 1.05 | 0.20 |
|  | Unknown | 1.04 | 0.87 | 1.24 | 0.69 |
| **Dual Enrolled** | Yes vs No | 1.12 | 0.96 | 1.30 | 0.16 |
| **Census Tract Poverty** | 0% -5% poverty (ref) |  |  |  |  |
|  | 5% -10% poverty | 1.04 | 0.89 | 1.22 | 0.60 |
|  | 10% -20% poverty | 1.03 | 0.87 | 1.20 | 0.76 |
|  | 20% -100% poverty | 0.84 | 0.68 | 1.03 | 0.10 |
|  | Unknown | 1.12 | 0.90 | 1.40 | 0.32 |
| **Metropolitan Residence** | Yes vs No | 0.98 | 0.83 | 1.16 | 0.84 |
| **ICI agent** | Pembrolizumab (ref) |  |  |  |  |
|  | Atezolizumab | 0.60 | 0.42 | 0.86 | 0.01 |
|  | Nivolumab | 0.91 | 0.79 | 1.03 | 0.14 |
| **Chemotherapy Combined with ICI** | Yes vs No | 0.87 | 0.73 | 1.08 | 0.23 |
| **Time Since Diagnosis** |  | 1.001 | 0.998 | 1.004 | 0.55 |
| **Cancer Stage** | Metastatic (ref) |  |  |  |  |
|  | Not metastatic | 1.10 | 0.97 | 1.24 | 0.151 |
| **Histology** | Adenocarcinoma (ref) |  |  |  |  |
|  | Large cell | 1.09 | 0.63 | 1.91 | 0.76 |
|  | Other/ not otherwise specified | 1.07 | 0.90 | 1.26 | 0.44 |
|  | Squamous cell | 1.09 | 0.96 | 1.24 | 0.21 |
| **Line Of Therapy For ICI** | 1L (ref) |  |  |  |  |
|  | 2L | 0.94 | 0.81 | 1.09 | 0.41 |
|  | 3L+ | 1.01 | 0.84 | 1.20 | 0.96 |
| **Radiation Before ICI** | Yes vs No | 1.17 | 1.03 | 1.32 | 0.01 |
| **Surgery Before ICI** | Yes vs No | 1.02 | 0.88 | 1.18 | 0.79 |
| **CNS Metastasis** | Yes vs No | 0.69 | 0.58 | 0.82 | <0.001 |
| **Autoimmune Disorder** | Yes vs No | 0.98 | 0.85 | 1.12 | 0.73 |
| **Disability Status** | Good (ref) |  |  |  |  |
|  | Poor | 1.04 | 0.93 | 1.17 | 0.50 |
| **Recent Use of Steroids** | Yes vs No | 0.99 | 0.88 | 1.11 | 0.89 |
| **NCI Comorbidity Index** |  | 1.12 | 0.99 | 1.27 | 0.06 |

Abbreviations: SHR, Subdistribution Hazard Ratio; CI, confidence interval; ICI, immune checkpoint inhibitors; 1L, first line; 2L, second line; 3L, third line.

**Table S6 Fine-Gray model for predicting the incidence of hypothyroidism**

| **Parameter** | | **Hypothyroidism** | | | |
| --- | --- | --- | --- | --- | --- |
|  |  | **SHR** | **95% CI** | | ***p*** |
| **Age** |  | 1.00 | 0.99 | 1.01 | 0.76 |
| **Sex** | Female (ref) |  |  |  |  |
|  | Male | 0.78 | 0.67 | 0.90 | <0.001 |
| **Race** | Black (ref) |  |  |  |  |
|  | White | 1.25 | 0.91 | 1.72 | 0.17 |
|  | Other | 1.05 | 0.66 | 1.67 | 0.83 |
| **Hispanic** | Yes vs No | 1.06 | 0.76 | 1.49 | 0.73 |
| **Region** | West (ref) |  |  |  |  |
|  | Midwest | 0.96 | 0.71 | 1.29 | 0.77 |
|  | Northeast | 1.67 | 1.38 | 2.03 | <0.001 |
|  | South | 1.03 | 0.81 | 1.31 | 0.81 |
| **Marital Status** | Live independently (ref) |  |  |  |  |
|  | Live with souse or partner | 1.03 | 0.87 | 1.23 | 0.71 |
|  | Unknown | 0.67 | 0.54 | 0.83 | <0.001 |
| **Dual Enrolled** | Yes vs No | 1.00 | 0.81 | 1.23 | 0.99 |
| **Census Tract Poverty** | 0% -5% poverty (ref) |  |  |  |  |
|  | 5% -10% poverty | 0.90 | 0.75 | 1.09 | 0.29 |
|  | 10% -20% poverty | 0.91 | 0.74 | 1.11 | 0.33 |
|  | 20% -100% poverty | 1.06 | 0.83 | 1.35 | 0.66 |
|  | Unknown | 1.08 | 0.82 | 1.42 | 0.60 |
| **Metropolitan Residence** | Yes vs No | 1.29 | 1.02 | 1.64 | 0.04 |
| **ICI Agent** | Pembrolizumab (ref) |  |  |  |  |
|  | Atezolizumab | 0.49 | 0.30 | 0.78 | 0.003 |
|  | Nivolumab | 0.76 | 0.64 | 0.89 | <0.001 |
| **Chemotherapy Combined With Ici** | Yes vs No | 0.87 | 0.69 | 1.11 | 0.26 |
| **Time Since Diagnosis** |  | 1.002 | 0.998 | 1.01 | 0.31 |
| **Cancer Stage** | Metastatic (ref) |  |  |  |  |
|  | Not metastatic | 1.17 | 0.995 | 1.37 | 0.06 |
| **Histology** | Adenocarcinoma (ref) |  |  |  |  |
|  | Large cell | 0.92 | 0.42 | 1.99 | 0.83 |
|  | Other/ not otherwise specified | 0.97 | 0.79 | 1.20 | 0.80 |
|  | Squamous cell | 1.04 | 0.88 | 1.23 | 0.67 |
| **Line Of Therapy for ICI** | 1L (ref) |  |  |  |  |
|  | 2L | 1.02 | 0.85 | 1.23 | 0.83 |
|  | 3L+ | 0.95 | 0.75 | 1.19 | 0.63 |
| **Radiation Before ICI** | Yes vs No | 1.01 | 0.86 | 1.17 | 0.95 |
| **Surgery Before ICI** | Yes vs No | 0.83 | 0.69 | 1.01 | 0.06 |
| **CNS Metastasis** | Yes vs No | 0.71 | 0.57 | 0.88 | 0.002 |
| **Autoimmune Disorder** | Yes vs No | 1.16 | 0.98 | 1.38 | 0.08 |
| **Disability Status** | Good (ref) |  |  |  |  |
|  | Poor | 0.88 | 0.76 | 1.02 | 0.09 |
| **Recent Use of Steroids** | Yes vs No | 0.98 | 0.84 | 1.14 | 0.78 |
| **NCI Comorbidity Index** |  | 0.82 | 0.69 | 0.97 | 0.02 |

Abbreviations: SHR, Subdistribution Hazard Ratio; CI, confidence interval; ICI, immune checkpoint inhibitors; 1L, first line; 2L, second line; 3L, third line.

**Table S7 Fine-Gray model for predicting the incidence of arrhythmia**

| **Parameter** | | **Arrhythmia** | | | |
| --- | --- | --- | --- | --- | --- |
|  |  | **SHR** | **95% CI** | | ***p*** |
| **Age** |  | 1.01 | 1.00 | 1.02 | 0.05 |
| **Sex** | Female (ref) |  |  |  |  |
|  | Male | 1.23 | 1.07 | 1.41 | 0.003 |
| **Race** | Black (ref) |  |  |  |  |
|  | White | 1.04 | 0.78 | 1.38 | 0.80 |
|  | Other | 0.89 | 0.58 | 1.36 | 0.60 |
| **Hispanic** | Yes vs No | 1.02 | 0.74 | 1.40 | 0.91 |
| **Region** | West (ref) |  |  |  |  |
|  | Midwest | 1.14 | 0.87 | 1.49 | 0.35 |
|  | Northeast | 1.19 | 0.98 | 1.45 | 0.09 |
|  | South | 1.07 | 0.86 | 1.34 | 0.55 |
| **Marital Status** | Live independently (ref) |  |  |  |  |
|  | Live with souse or partner | 1.04 | 0.88 | 1.23 | 0.68 |
|  | Unknown | 1.11 | 0.90 | 1.37 | 0.33 |
| **Dual Enrolled** | Yes vs No | 1.16 | 0.96 | 1.40 | 0.13 |
| **Census Tract Poverty** | 0% -5% poverty (ref) |  |  |  |  |
|  | 5% -10% poverty | 1.01 | 0.83 | 1.22 | 0.95 |
|  | 10% -20% poverty | 1.06 | 0.87 | 1.29 | 0.56 |
|  | 20% -100% poverty | 1.08 | 0.85 | 1.37 | 0.55 |
|  | Unknown | 1.12 | 0.86 | 1.47 | 0.39 |
| **Metropolitan Residence** | Yes vs No | 1.07 | 0.87 | 1.32 | 0.51 |
| **ICI Agent** | Pembrolizumab (ref) |  |  |  |  |
|  | Atezolizumab | 1.00 | 0.70 | 1.45 | 0.99 |
|  | Nivolumab | 0.92 | 0.79 | 1.08 | 0.30 |
| **Chemotherapy Combined with ICI** | Yes vs No | 0.99 | 0.79 | 1.24 | 0.93 |
| **Time Since Diagnosis** |  | 1.00 | 0.999 | 1.01 | 0.10 |
| **Cancer Stage** | Metastatic (ref) |  |  |  |  |
|  | Not metastatic | 0.98 | 0.84 | 1.15 | 0.83 |
| **Histology** | Adenocarcinoma (ref) |  |  |  |  |
|  | Large cell | 0.58 | 0.24 | 1.39 | 0.22 |
|  | Other/ not otherwise specified | 1.21 | 0.99 | 1.46 | 0.06 |
|  | Squamous cell | 1.09 | 0.93 | 1.28 | 0.30 |
| **Line of Therapy For ICI** | 1L (ref) |  |  |  |  |
|  | 2L | 0.86 | 0.73 | 1.02 | 0.08 |
|  | 3L+ | 0.73 | 0.58 | 0.92 | 0.01 |
| **Radiation Before ICI** | Yes vs No | 0.96 | 0.83 | 1.11 | 0.56 |
| **Surgery Before ICI** | Yes vs No | 0.94 | 0.78 | 1.13 | 0.49 |
| **CNS Metastasis** | Yes vs No | 0.86 | 0.71 | 1.06 | 0.16 |
| **Autoimmune Disorder** | Yes vs No | 0.99 | 0.84 | 1.17 | 0.91 |
| **Disability Status** | Good (ref) |  |  |  |  |
|  | Poor | 1.06 | 0.92 | 1.22 | 0.43 |
| **Recent Use of Steroids** | Yes vs No | 0.97 | 0.84 | 1.12 | 0.68 |
| **NCI Comorbidity Index** |  | 0.95 | 0.81 | 1.11 | 0.50 |

Abbreviations: SHR, Subdistribution Hazard Ratio; CI, confidence interval; ICI, immune checkpoint inhibitors; 1L, first line; 2L, second line; 3L, third line.

**Table S8** **Fine-Gray model for predicting the incidence of AKI**

| **Parameter** | | **AKI** | | | |
| --- | --- | --- | --- | --- | --- |
|  |  | **SHR** | **95% CI** | | ***p*** |
| **Age** |  | 1.01 | 0.997 | 1.03 | 0.10 |
| **Sex** | Female (ref) |  |  |  |  |
|  | Male | 1.22 | 0.999 | 1.48 | 0.051 |
| **Race** | Black (ref) |  |  |  |  |
|  | White | 0.66 | 0.46 | 0.93 | 0.02 |
|  | Other | 0.75 | 0.45 | 1.23 | 0.25 |
| **Hispanic** | Yes vs No | 1.10 | 0.71 | 1.73 | 0.67 |
| **Region** | West (ref) |  |  |  |  |
|  | Midwest | 1.45 | 1.03 | 2.05 | 0.04 |
|  | Northeast | 1.01 | 0.78 | 1.30 | 0.96 |
|  | South | 1.07 | 0.78 | 1.47 | 0.66 |
| **Marital Status** | Live independently (ref) |  |  |  |  |
|  | Live with souse or partner | 1.13 | 0.89 | 1.44 | 0.30 |
|  | Unknown | 1.25 | 0.94 | 1.67 | 0.13 |
| **Dual Enrolled** | Yes vs No | 1.43 | 1.09 | 1.87 | 0.01 |
| **Census Tract Poverty** | 0% -5% poverty (ref) |  |  |  |  |
|  | 5% -10% poverty | 0.78 | 0.61 | 1.01 | 0.06 |
|  | 10% -20% poverty | 0.75 | 0.58 | 0.98 | 0.03 |
|  | 20% -100% poverty | 0.64 | 0.46 | 0.90 | 0.01 |
|  | Unknown | 0.68 | 0.46 | 0.999 | 0.049 |
| **Metropolitan Residence** | Yes vs No | 1.13 | 0.83 | 1.54 | 0.43 |
| **ICI Agent** | Pembrolizumab (ref) |  |  |  |  |
|  | Atezolizumab | 0.78 | 0.47 | 1.31 | 0.36 |
|  | Nivolumab | 0.64 | 0.52 | 0.80 | <0.001 |
| **Chemotherapy Combined with ICI** | Yes vs No | 0.98 | 0.72 | 1.32 | 0.87 |
| **Time Since Diagnosis** |  | 1.00 | 0.996 | 1.01 | 0.50 |
| **Cancer Stage** | Metastatic (ref) |  |  |  |  |
|  | Not metastatic | 0.95 | 0.76 | 1.18 | 0.63 |
| **Histology** | Adenocarcinoma (ref) |  |  |  |  |
|  | Large cell | 0.67 | 0.22 | 2.05 | 0.48 |
|  | Other/ not otherwise specified | 0.84 | 0.62 | 1.13 | 0.24 |
|  | Squamous cell | 0.83 | 0.66 | 1.04 | 0.10 |
| **Line Of Therapy for ICI** | 1L (ref) |  |  |  |  |
|  | 2L | 1.00 | 0.79 | 1.27 | 0.99 |
|  | 3L+ | 0.90 | 0.66 | 1.24 | 0.52 |
| **Radiation Before ICI** | Yes vs No | 0.94 | 0.76 | 1.16 | 0.53 |
| **Surgery Before ICI** | Yes vs No | 0.94 | 0.73 | 1.21 | 0.63 |
| **CNS Metastasis** | Yes vs No | 0.74 | 0.55 | 0.99 | 0.05 |
| **Autoimmune Disorder** | Yes vs No | 1.20 | 0.97 | 1.50 | 0.10 |
| **Disability Status** | Good (ref) |  |  |  |  |
|  | Poor | 0.92 | 0.75 | 1.11 | 0.38 |
| **Recent Use of Steroids** | Yes vs No | 1.15 | 0.94 | 1.41 | 0.19 |
| **NCI Comorbidity Index** |  | 1.58 | 1.31 | 1.89 | <0.001 |

Abbreviations: SHR, Subdistribution Hazard Ratio; CI, confidence interval; ICI, immune checkpoint inhibitors; 1L, first line; 2L, second line; 3L, third line.
